# A Magnetoresistive Tactile Sensor for Harsh Environment Applications

**DOI:** 10.3390/s16050650

**Published:** 2016-05-07

**Authors:** Ahmed Alfadhel, Mohammed Asadullah Khan, Susana Cardoso, Diana Leitao, Jürgen Kosel

**Affiliations:** 1 Computer, Electrical and Mathematical Sciences and Engineering Division (CEMSE), King Abdullah University of Science and Technology (KAUST), Thuwal 23955-6900, Saudi Arabia; ahmed.fadhel@kaust.edu.sa (A.A.); mohammedasadullah.khan@kaust.edu.sa (M.A.K.); 2 INESC-Microsystems and Nanotechnologies (INESC-MN), Rua Alves Redol, 9, Lisbon 1000-029, Portugal; scardoso@inesc-mn.pt (S.C.); dleitao@inesc-mn.pt (D.L.); 3 Instituto Superior Técnico IST, Physics Department, Universidade de Lisboa, Lisbon 1049-001, Portugal

**Keywords:** magnetic nanocomposite, giant magnetoresistance, high temperature, harsh environment, nanowires, cilia, tactile sensor, spin-valve

## Abstract

A magnetoresistive tactile sensor is reported, which is capable of working in high temperatures up to 140 °C. Hair-like bioinspired structures, known as cilia, made out of permanent magnetic nanocomposite material on top of spin-valve giant magnetoresistive (GMR) sensors are used for tactile sensing at high temperatures. The magnetic nanocomposite, consisting of iron nanowires incorporated into the polymer polydimethylsiloxane (PDMS), is very flexible, biocompatible, has high remanence, and is also resilient to antagonistic sensing ambient. When the cilia come in contact with a surface, they deflect in compliance with the surface topology. This yields a change of the GMR sensor signal, enabling the detection of extremely fine features. The spin-valve is covered with a passivation layer, which enables adequate performance in spite of harsh environmental conditions, as demonstrated in this paper for high temperature.

## 1. Introduction

Advances in computing and artificial intelligence have resulted in increasingly capable robotic systems that have the ability of delivering revolutionary advances in the fields of healthcare, remote search and rescue operations, deep sea exploration, mining, *etc*. However, in order to enable these applications, the robots need a plethora of precise sensors, among which tactical sensors with excellent spatial and pressure resolution are of particular importance [1,2,3,4]. In addition to robots, tactical sensors can enhance, for example, prosthetic limbs, artificial skin, and surgical instruments [5,6].

Various tactile pressure sensors have been presented in literature that use different principles for transduction. For example, [7] presents a self-powered tactile sensor. This sensor consists of a polymer polydimethylsiloxane (PDMS)/Indium Tin Oxide (ITO) textured micro-pyramid sheet and utilizes triboelectric charging to generate a voltage signal that is proportional to the applied stress. This sensor is capable of 400 Pa resolution over a range of 0 to 7.3 kPa. In [8], the deformation of a pyramidal textured PDMS membrane deformed by tactile input is used to scatter light in an acrylic waveguide, which is then captured by a camera and used to determine the stress on the PDMS membrane. This sensor is capable of registering touches as light as 1 kPa over a range of −60 kPa to 60 kPa. A resistive tactile sensor, in which the change in resistance of a Molybdenum Sulfide layer deposited on a SU-8 substrate serves as the transduction mechanism, is reported in [9]. This sensor operates linearly between 0 to 100 kPa with a minimum detection threshold of 1.24 kPa.

Polymeric artificial cilia on a magnetic sensor have been presented previously [10]. Polypyrole cilia have been fabricated on top of giant magnetoresistive (GMR) sensors and were tagged with a 300 nm Co_50_Fe_50_ by sputter deposition. The sensor was tested by agitation at different frequencies and measuring the response due to the cilia bending. The proposed design suffers from several issues such as the easy corrosion of the magnetic tagging, the toxicity of Co, the low stray field expected from the magnetic tagging, and the non-uniform tagging that lead to having cilia with different magnetic and mechanical properties.

However, as capable as the above sensors are, they have not been tested in harsh environmental conditions. Thus, their efficacy, for instance, in high temperature environments is unproven, which is quite important because some tasks, such as fire rescue operations or industrial work, need tactile sensors that can work in adverse conditions. There are conventional pressure sensors that have been tested at high temperatures, however, they are not suitable for tactile applications. For example, [11] uses a low-temperature cofireable ceramic (LTCC) process to make a wireless passive pressure sensor that can work at temperatures up to 400 °C with linear sensitivity between 0 and 700 kPa. Being made of rigid ceramic it is not conformal and its resolution of 2.4 kPa is more suited for high pressure applications than for sensing touch. A high temperature pressure sensor, which uses a SiC membrane and capacitive transduction to achieve a resolution of 5.2 kPa in the range 147–235 kPa, is demonstrated in [12]. While capable of operating at temperatures up to 400 °C, its sensing range and resolution are not good enough to meet the requirement for typical tactile applications. A piezoresistive pressure sensor made on silicon on insulator (SoI) substrate that operates at temperatures up to 500 °C with an ability to resolve pressures up to 20 kPa is reported in [13]. It is not suited for large area applications like artificial skin due to the high cost of the SoI substrate and also the resolution is not suited for sensing gentle touches and textures.

Polymer composites have gained attention as potential candidates for sensing and energy harvesting as they offer various benefits such as ease of manufacture, tunable properties, mechanical flexibility, low cost, low toxicity, and conformality. Depending upon the doping material and the polymer itself, the composite can be conductive, piezoelectric, triboelectric, magnetic, *etc.* [14,15,16,17,18,19,20,21,22,23]. Composites have been made using super paramagnetic nanobeads for various applications. However, since superparamagnetic particles have very low remanent magnetization, they require an externally generated magnetic field for operation, which greatly reduces power efficiency and portability.

For our tactical sensor we use a magnetic nanocomposite comprising of the polymer PDMS and iron nanowires (NWs). PDMS has very low stiffness, is chemically resistant, and is biocompatible. Due to the large shape anisotropy, iron NWs exhibit high remanent magnetization. Thus, this magnetic nanocomposite exhibits large remanent magnetization and flexibility. Iron NWs are also not cytotoxic [24] and thus the composite is suitable for medical applications. Typical electrodeposited iron NWs are polycrystalline which can be oxidized if exposed to the environment, reducing the magnetization, and hence, degrading the sensor’s performance. This has been studied previously via corroding polycrystalline iron NWs by oxidizing them through annealing at 150 °C [24]. The formation of a magnetite shell around an iron core was observed, which happened by oxygen diffusion between the grain boundaries of iron NWs, starting from the surface of the NWs. The iron core’s diameter decreased as the oxide shell thickness increased until the NWs were fully oxidized, yielding a polycrystalline magnetite structure. As a result, only 40% of the magnetization was maintained by the magnetite NWs [24]. In this work, the issue of corrosion is addressed by embedding the polycrystalline iron NWs inside of a polymer, which provides a high level of protection.

The reported sensor consists of a 3 × 3 array of 1 mm long, 200 µm wide cilia made up of the magnetic nanocomposite mounted on top of a 37 × 16 array of spin-valve elements connected in series (total of 592 elements). It occupies an area of 1 mm × 1 mm. Due to the low Young’s modulus and large aspect ratio, the cilia are sensitive to both in-plane as well as out of plane mechanical forces. This causes them to respond with significant deflection to even the lightest of touch. This sensor is also capable of vibration and fluid flow detection. As the electrical connections to the spin-valve elements are not affected by the mechanical forces applied, the sensor is robust. Unlike conventional capacitive sensors, this sensor can be used both in dry and wet conditions. It is also capable of studying both dynamic and static forces.

This paper builds upon a previously reported tactile sensor [17,18] and flow sensor [20], which uses a similar transduction mechanism but with a giant magnetoimpedance (GMI) sensor to detect the stray field of the cilia. The GMI sensor needs a high frequency signal for operation, which can prove to be an impediment for certain use case scenarios. In this work, spin-valve GMR sensors are utilized as they can be operated using direct current (DC) signals. This in turn simplifies the circuitry and instrumentation needed for the measurement. The effect of temperature on the performance of the spin-valve elements and the nanocomposite is evaluated and the overall ability of the tactile sensor to withstand high temperature conditions is gauged.

## 2. Materials and Methods

### 2.1. Tactile Sensing Concept

**Figure 1:**
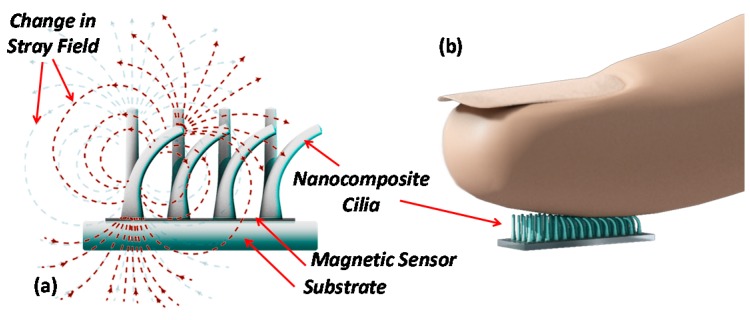
(**a**) Illustration of the tactile sensor working principle. In the standing position, the cilia’s stray magnetic field affects the spin-valve giant magnetoresistive (GMR) sensor and biases it to an initial resistance value. In the presence of an external force, the cilia bend and the stray field measured with the spin-valve changes, resulting in a change in the resistance; (**b**) Example of the cilia deflection mechanism when touched with a finger.

The magnetic tactile sensing concept utilizes permanent magnetic and highly elastic nanocomposite cilia integrated on a magnetic sensing element. A magnetic sensor detects the change of the magnetic stray field, created by the permanent magnetic cilia, when deflected by an external force (Figure 1). A spin-valve GMR sensor, which is simple to fabricate and can be applied onto flexible substrates, is utilized to measure the change in the magnetic field [25,26,27]. When the cilia are standing in the rest position, their stray field biases the GMR sensor. In the presence of an external force the cilia bend, resulting in a change of the stray field; hence, changing the sensor’s resistance.

The tactile sensor consists of 3 × 3 cilia applied on top of a spin-valve array connected in series forming an active area of 1 × 1 mm^2^. The effective pressure is quantified by calculating the ratio of the applied forces and the active area. Each spin-valve is 60 μm long and 3 μm wide with sensitivity along the width direction.

The artificial cilia deflection due to an external force can be described by the Euler–Bernoulli beam theory (classical beam theory) [20,28]. Using the displacement-force relationship of a cylindrical beam, the displacement *δ* can be quantified when applying a force *F* at the free end of a beam with length *l*, Young’s modulus *E*, and diameter *D*, such as:

(1) $$\delta =\vec{F}\frac{64l^{3}}{3\pi ED^{4}}$$

### 2.2. Fabrication

**Figure 2:**
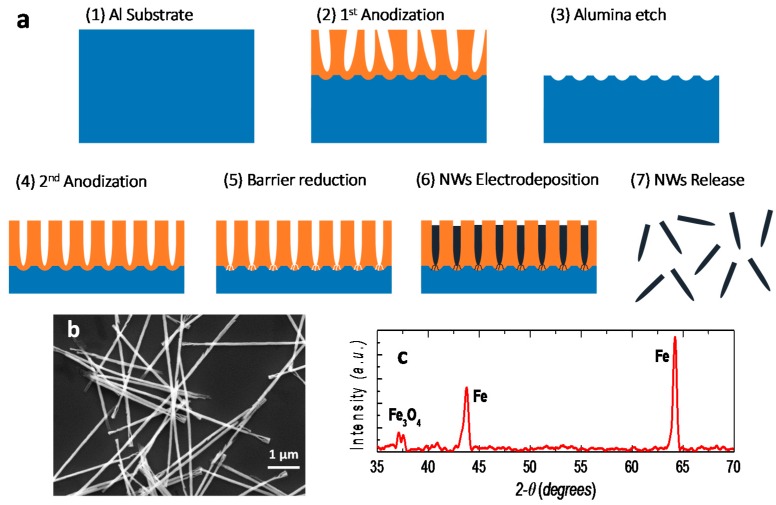
(**a**) Illustration of the electrochemical fabrication process of iron nanowires (NWs). (1) A nanoporous template is formed using an aluminum substrate. (2–4) The aluminum is anodized to create nanoporous alumina in two steps, with an alumina etching step in between to obtain uniform nanopores of ~35 nm in diameter. (5) A barrier reduction step establishes an electrical contact with the bottom electrode. (6) Iron is electrodeposited into the nanoporous membrane. (7) The NWs are released by etching the alumina membrane and dispersed in ethanol; (**b**) Scanning electron microscopy image of released NWs; (**c**) X-ray Diffraction (XRD) result of iron NWs.

Iron NWs were fabricated by the electrodeposition into a nanoporous aluminum oxide membrane, a well-known technology to fabricate dense array with high aspect ratio NWs (Figure 2a). The aluminum oxide membrane with dense hexagonally patterned nanopores was prepared by a two-step anodization process, which consisted of an electrochemical oxidation of aluminum substrate using oxalic acid and with controlled temperature and electrical potential [29]. After electrodepositing NWs into the membranes, they were released and dispersed in ethanol. Six micrometer long iron NWs with a diameter of 35 nm were fabricated to act as nanoscale permanent magnets. The dimensions and morphology of released iron NWs were investigated using scanning electron microscopy (Figure 2b). X-ray Diffraction (XRD) experiments were carried out, using a Bruker D8 Discover high-resolution X-ray diffractometer system that provides X-ray radiation with wavelength of 1.5406 Å, to determine the NWs composition and crystal structure. The XRD spectrum in Figure 2c reveals that the NWs are polycrystalline iron (Fe) and magnetite (Fe_3_O_4_), which is growing as a thin shell around the NWs. Using various characterizations, the same type of NWs has been studied before confirming the iron/magnetite core-shell structure [24].

The magnetic nanocomposite was synthesized by washing dispersed iron NWs with Sodium dodecyl sulfate (SDS) surfactant, and then mixing them with PDMS (Sylgard 184 Silicone Elastomer, Dow Corning Corporation, Midland, MI, USA). The nanocomposite was then cured, whereby the NWs concentration can be adjusted easily. A NWs to PDMS volume ratio of 14% was chosen to reach a high magnetization of the nanocomposite while avoiding adverse effects on the polymerization process of the PDMS or the elasticity of the cilia.

**Figure 3:**
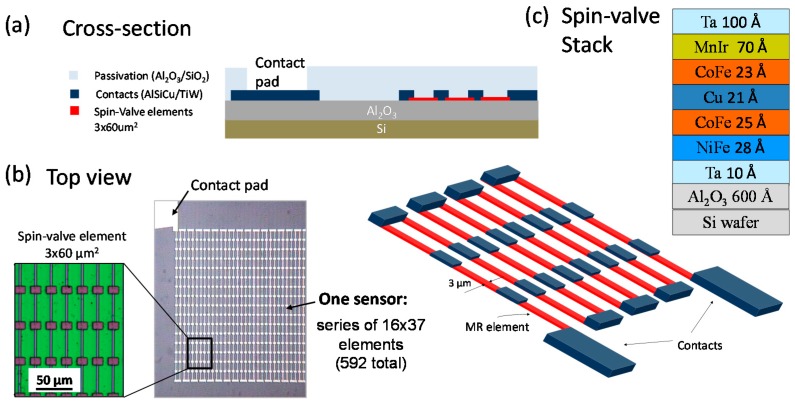
GMR sensor layout and geometry: (**a**) Cross section of the spin-valve sensor array connected in series; and (**b**) Top view of the samples microfabricated; and (**c**) the spin-valve thin films stack.

The magnetic sensing element is a spin-valve sensor, microfabricated on a 150 mm diameter Si (100) wafer passivated with 60 nm of Al_2_O_3_ deposited by RF magnetron sputtering. Top pinned spin valve sensors were fabricated by ion beam deposition (Nordiko3600 system, from Nordiko Tech. Services, Hampshire, England) with the following structure (thickness in nm): substrate/Ta(1.0)/Ni_80_Fe_20_(2.8)/Co_90_Fe_10_(2.5)/Cu(2.1)/Co_90_Fe_10_(2.3)/Mn_78_Ir_22_(7.0)/Ta(10.0) [30] (compositions in at%). A 5 mT magnetic field was applied during deposition to induce the free layer and pinned layer easy axis with parallel anisotropies. The sensors were defined by direct write laser lithography (DWL) and by ion milling (Nordiko 3000 system). The electrical leads were patterned by DWL in a 2-contact geometry, and consist of 300 nm sputtered Al (Al_98.5_Si_1_Cu_0.5_ in at%) and 15 nm of TiW(N_2_) films defined by liftoff. The sensors consist of arrays of 592 rectangular spin-valve elements (each with a sensing area of 3 μm × 60 μm) connected in series and occupying a 1 × 1 mm^2^ total area. The geometry was optimized to minimize the sensor signal-to-noise level [31], because of the total volume of magnetic material, while maintaining a well-defined linearity in the transfer curve [32]. The sensor was passivated with sputtered Al_2_O_3_ (200 nm) and SiO_2_ (200 nm) deposited by magnetron sputtering (final passivation layer) to protect against corrosion and to enable the chemical bonding with the PDMS [33]. The fabrication details are illustrated in Figure 3.

**Figure 4:**
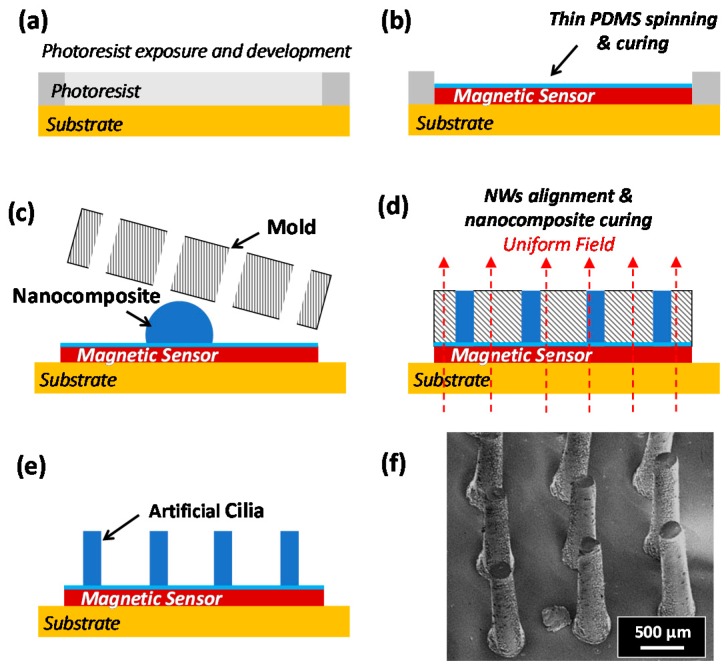
Illustration of the tactile sensor fabrication process. (**a**,**b**) Spin-valves were fabricated using standard lithography process, ion beam deposition and ion milling on a silicon substrate. A thin polydimethylsiloxane (PDMS) layer was then spun and cured; (**b**–**e**) A poly(methylmethcrylate) (PMMA) mold technique was used for the fabrication of nanocomposite cilia. A homogeneous magnetic field was applied during the curing process to align the NWs. The mold is peeled off after curing the nanocomposite resulting in cilia mounted on the magnetic sensor; (**f**) Scanning Electron Microscopy image of the cilia array, where each cilium is 1 mm in length and 200 μm in diameter.

A 5 μm PDMS layer was spun on top of the magnetic sensor to provide electrical isolation and, at the same time, enhance the adhesion of the cilia to the spin-valve. A master mold technique was utilized to pattern the cilia. The master mold is a 1 mm thick poly(methylmethcrylate) (PMMA) with 200 μm in diameter 3 × 3 array of holes patterned using a CO_2_ laser cutter (Universal PLS6.75). The nanocomposite is casted onto the surface of the thin PDMS/spin-valve stack, and the master mold is mounted on top. A uniform magnetic field along the cilia is applied using a C-shaped magnet. This structure is then placed in a desiccator for 30 min to remove any trapped air bubbles and assist in filling the pores. Next, the composite is cured at 90 degrees Celsius for one hour, forming the cilia with aligned NWs on top of the spin-valve. After releasing the cured cilia, they are annealed at 150 °C to increase the Young’s modulus and to withstand high temperature operation without significant variation in the material’s elasticity, and hence, the operating range. The cilia are then fully magnetized by applying a magnetic field of 10 kOe. The fabrication process is illustrated in Figure 4.

### 2.3. Characterization

The magnetic properties of the nanocomposite cilia were studied to confirm the permanent magnetic behavior. The magnetization loops along the cilia in the parallel and perpendicular directions were obtained using a vibrating sample magnetometer (VSM) (PMC MicroMag 3900, Westerville, OH, USA, and superconducting quantum interference device (SQUID) VSM 1500-100 by Quantum design Inc., San Diego, CA, USA).

In order to characterize the spin-valves array, a Helmholtz coil was used to apply a homogeneous magnetic field along the sensitive and non-sensitive directions, and the resistance was measured directly with a source meter (Keithley 2400) at a DC current of 100 µA.

The high elasticity of the nanocomposite is crucial to obtain a high sensitivity with the tactile sensor. Therefore, a study was conducted to investigate if adding NWs to the PDMS affects the elasticity. The Young’s modulus of the nanocomposite cilia was obtained by fixing them between the holder plate and the moveable head of an extensometer tensile/compressive force testing system (INSTRON 5966). The system can precisely determine the exerted force and the vertical displacement. By using a 10 N load cell and three cilia that are 1 mm long and 200 µm in diameter, resolutions of 5 mN and 16 µm were obtained for the tensile force and vertical displacement, respectively. Tensile forces were applied to the cilia until achieving a strain of 1.3 (130% stretching). The Young’s modulus was calculated as the ratio between stress and strain (slope) in the elastic region.

**Figure 5:**
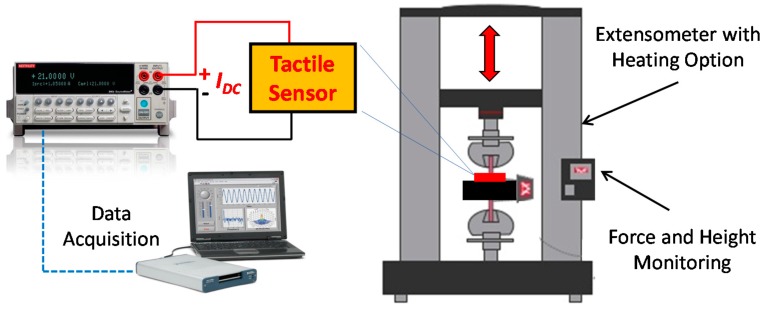
Experimental setup for the tactile sensor testing.

The performance of the tactile sensor was studied by applying vertical forces at different temperatures while measuring the sensor response. The forces were applied using the computer controlled force testing system, which can precisely determine the amount of force applied to the sensors. The system comes with an oven that allows setting the ambient temperature during the experiment. The resistance variation upon the deflection of the cilia was obtained directly using a source meter (Keithley 2400) that can apply a fixed current value throughout the measurement. The experimental setup is illustrated in Figure 5.

## 3. Results and Discussion

### 3.1. Nanocomposite Characterization

**Figure 6:**
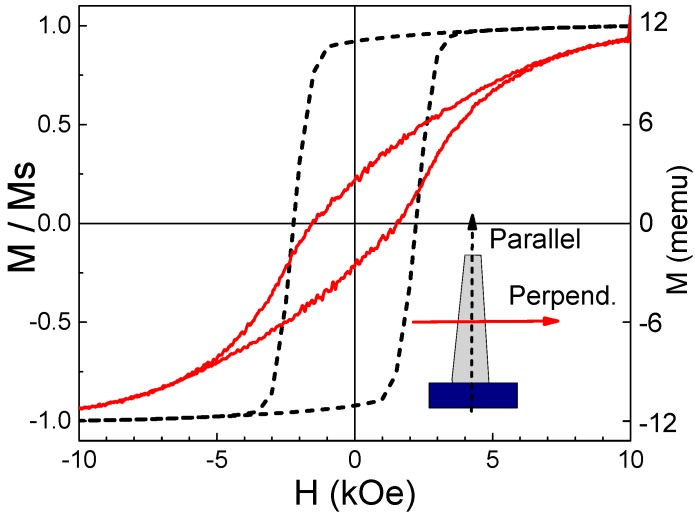
Nanocomposite magnetization loops obtained for parallel and perpendicular direction of a cilia array. The NWs in the cilia are aligned in the parallel direction.

The magnetic properties of the nanocomposite cilia were obtained by measuring the magnetizations loops shown in Figure 6. The cilia with NWs aligned along the parallel direction had a magnetic anisotropy with a saturation magnetization (*Ms*) of 11.7 memu, and remanence to saturation magnetization (*Mr/Ms*) of 93% and 19% along the parallel and perpendicular direction, respectively. The coercivity along the parallel direction was found to be 2.1 kOe, and coercivity along the perpendicular direction was 1.6 kOe. These results confirm both the permanent magnetic behavior of the nanocomposite and the alignment of the NWs.

**Figure 7:**
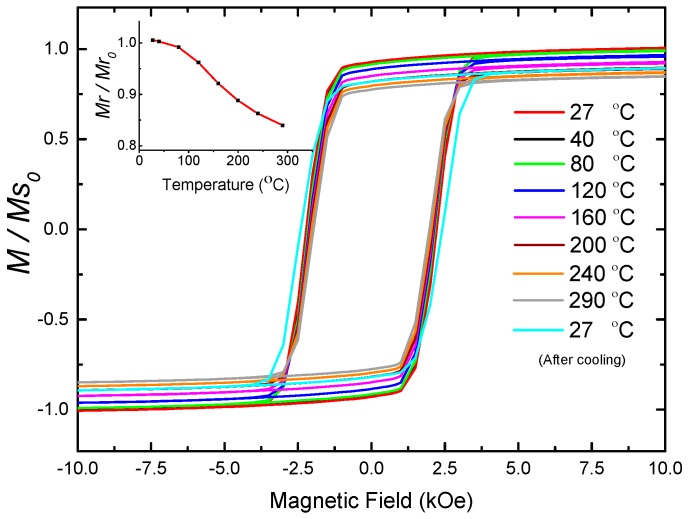
Magnetization loops of nanocomposite cilia at different temperatures from 27 °C to 290 °C, showing the magnetization *M* normalized to the initial saturation magnetization (*Ms_0_*) at 27 °C. Inset: remanence magnetization (*Mr*) as function of temperature normalized to initial remanence magnetization (*Mr_0_*) at 27 °C.

NWs exhibit very promising magnetic properties, suggesting that they could be used as permanent magnets that can operate at high temperatures. Through their strong shape anisotropy, NWs exhibit a large temperature independent coercivity when operating below the Curie temperature, which is 770 °C for iron [34]. Hence, the high magnetization of iron can be utilized while remaining below the Curie temperature. On the other hand, we have shown that the polycrystalline NWs are prone to corrosion [24]. In order to elucidate the thermal stability of the nanocomposite, where the NWs are protected by the polymer, we conducted a temperature dependent study of the magnetization. The hysteresis loops of a 1 mm long and 200 μm in diameter cilia were obtained at a temperature range between 27 °C and 290 °C using SQUID VSM. The cilia were heated in a vacuum oven during the magnetization measurement, and the hysteresis loops were obtained for each temperature value. Within this temperature range, the permanent magnetic behavior of the nanocomposite was maintained, as shown in Figure 7, where only a slight magnetization reduction was observed as a result of Curie’s law. The nanocomposite was then cooled down to 27 °C and the magnetization loop was measured again revealing a reduction in the remanence magnetization *Mr* of 16% (compared to the initial remanence magnetization, *Mr_0_*, at 27 °C) with an increase in coercivity compared to the initial state. The reduction in remanence can be caused by trapped water molecules in the PDMS that resulted in slightly oxidizing the NWs in the nanocomposite with the heat assisted oxygen diffusion process, forming a thin shell of magnetite around the NWs. The magnetite shell reduces the diameter of the iron core in the NWs, increasing its coercivity, due to the increased aspect ratio.

As can be seen from Equation (1), the Young’s Modulus affects the sensor signal, since a change in the Modulus changes the cilia deflection. Hence, we investigated the effect of NWs on the nanocomposite’s stiffness. The results of the tensile/compressive force tests show that nanocomposite cilia have a Young’s modulus of 1.1 MPa, compared to the pure PDMS cilia that have a Young’s modulus of 1.04 MPa. Thus, incorporating NWs in the nanocomposite does not have a significant effect on the rigidity of the cilia, and the advantage of the high elasticity of the PDMS is maintained.

### 3.2. GMR Sensor Response and Thermal Stability

**Figure 8:**
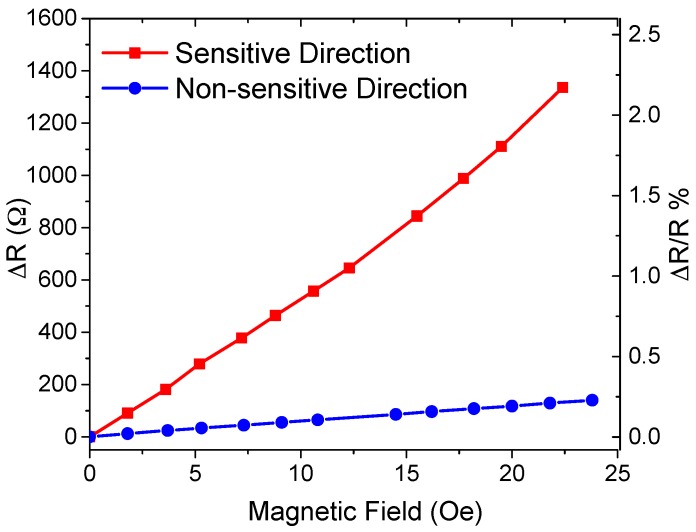
Resistance change ΔR (left axis) and magnetoresistive ratio (right) of the GMR sensor array with an external magnetic field applied to the sensitive and non-sensitive directions.

The spin-valve GMR sensor array showed a linear response to a magnetic field between 0 and 23 Oe with sensitivities of 57 Ω/Oe and 4.8 Ω/Oe in the sensitive and non-sensitive directions, respectively (Figure 8). The average magnetoresistive (MR) sensitivity was 0.08%/Oe and 0.01%/Oe in the sensitive (along the width of the GMR elements) and non-sensitive directions (along the length of the GMR elements), respectively, when considering the sensor’s initial resistance at room temperature, which is 62 kΩ. This anisotropic behavior equips the cilia sensor array with a directional sensitivity.

**Figure 9:**
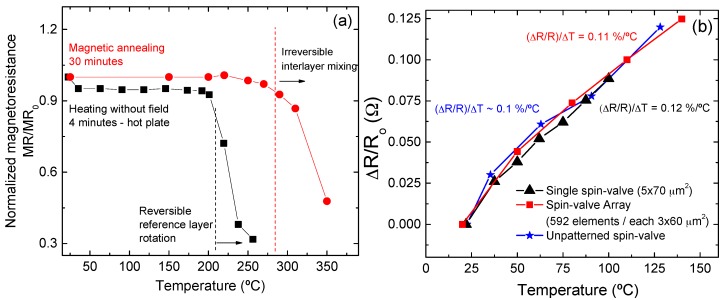
(**a**) Magnetoresistance dependence on temperature when a magnetic field is applied (magnetic annealing) and upon heating for 4 min on a hot plate without field. The data is normalized by the MR value at room temperature, measured before the thermal treatments; (**b**) Resistance dependence on temperature, tested for un-patterned spin-valve, a single microfabricated spin-valve, and the full sensor array, showing a similar dependency with variation of about 0.1%/°C.

The thermal stability of the GMR sensor was evaluated under two situations: (i) after magnetic annealing, measuring the MR response after 30 min of consecutive annealing steps up to 350 °C, with a 3 kOe applied field during cooling; and (ii) after heating 4 min on a hot plate without a magnetic field applied. Results are plotted in Figure 9a. When heated and cooled under a magnetic field, the MR signal is stable upon annealing up to 280 °C, which is the onset for the irreversible diffusion among the metallic layers, and is consistent with previous results obtained with CoFe/MnIr-based magnetoresistive stacks [35]. On the contrary, if the sample is heated without a magnetic field above 200 °C (close to the blocking temperature of the MnIr antiferromagnet [36]), the reference layer and pinned layer will rotate coherently and the MR signal is rapidly reduced. This, however, is a reversible process, and the sample can recover the full MR level upon annealing under a magnetic field to set the exchange coupling between the CoFe/MnIr interfaces. Figure 9b shows the resistance change (ΔR) of spin-valve sensors normalized to the initial resistance (R_0_) (at room temperature). Three different sensors were tested including: an un-patterned spin-valve, a single microfabricated spin-valve, and the full sensor array used in this work. (ΔR/R_o_) is a linear function of temperature change ΔT with a slope of ~0.1%/°C. The signal does not depend on the sensors overall resistance, shape or size.

### 3.3. Tactile Sensor Response

**Figure 10:**
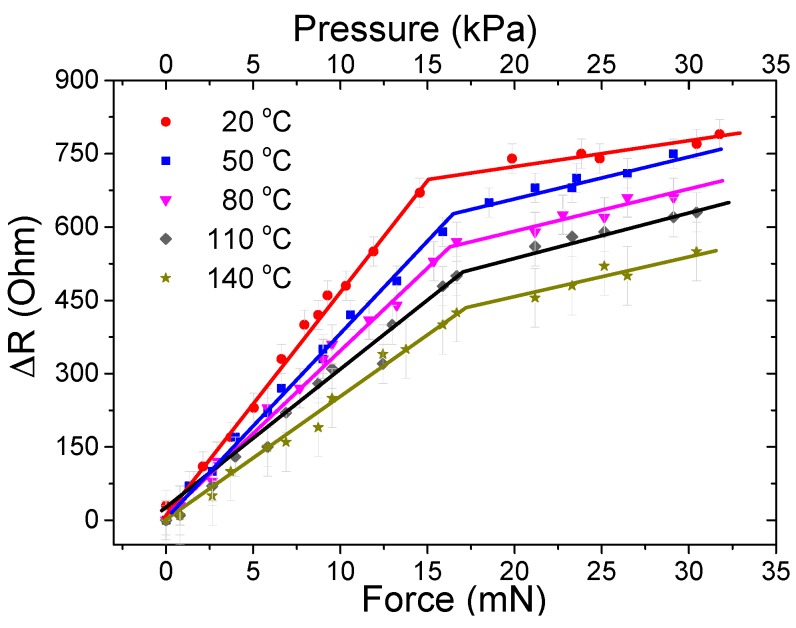
Tactile sensor response between 20 and 140 °C for different vertical forces/pressures. Pressures are calculated by considering the forces acting on the sensor’s active area.

The tactile sensor was tested by applying vertical forces up to 32 mN (32 kPa) at different temperatures between 20 and 140 °C, and the variation in resistance was recorded. The cilia sensor response to external forces is shown in Figure 10. The results at room temperature show the sensor’s ability to detect forces up to 15 mN (15 kPa) with a high sensitivity of 46 Ω/mN (46 Ω/kPa) and with noise of ±30 Ω. Considering this noise level, the resolution is 1.3 mN. Beyond this high sensitivity range, another linear region is found where the signal starts saturating, thereby providing a lower sensitivity. This region corresponds to the nearly full deflection of the cilia. The results for the tactile sensor at the high sensitivity region when operated at elevated temperatures are shown in Table 1. As the temperature increases, the signal exhibits lower resistance changes, which is attributed to the reduction in the nanocomposite’s magnetization. It is observed that the nanocomposite is mechanically stable, but undergoes a small reduction in elasticity which slightly hardens the nanocomposite, increases the Young’s modulus, and slightly increases the operating range. Previous studies have shown that the mechanical properties are maintained at low heating temperatures. Operating at temperatures greater than 200 °C will reduce the mechanical strength due to the thermal decomposition of PDMS that starts at about 200 °C and peaks at around 310 °C [37].

The magnetization is reduced from both Curie’s law and the slight corrosion of the iron NWs due to the heat assisted oxygen diffusion in the NWs. The latter is attributed to trapped water molecules in the PDMS, leading to corrosion of the NWs [20]. This is in accordance with a previous study [20] in which bare NWs were exposed to air, a nanocomposite sample was exposed to air, and another nanocomposite sample was kept in water. It was found that PDMS offers a good level of protection, and the magnetization of NWs inside of it slightly drops over time. After 10 days, the remanent magnetization of bare NWs in air dropped to 85% of the initial magnetization compared to 98% and 92.5% of nanocomposites exposed to air and water, respectively. NWs inside the nanocomposite oxidized slowly, due to the fact that PDMS is permeable to water molecules allowing minor interaction between iron and oxygen.

The reduction rate in sensitivity was −0.175 (Ω/mN)/°C, and the reduction in the signal measured at 15 mN (15 kPa) was −2.6 Ω/°C. The operating range was increasing at higher temperatures, which was attributed to the slight stiffening in the nanocomposite cilia. An expected increase in the signal variation was obtained, due to the increase of thermal noise, which as a result reduced the resolution.

## 4. Conclusions

A NWs-based permanent magnetic and highly elastic nanocomposite cilia tactile sensor has been developed for operation at elevated temperatures. Spin-valves detect the change of the stray field created by the permanent magnetic cilia when deflected due to an external force. The concept does not require an external magnetic field; hence the sensors can be highly integrated and employed in different applications without the added bulkiness of electro or permanent magnets.

The tactile sensor performance and operational abilities at high temperature were investigated. The effects of high temperatures on the nanocomposite are a small reduction in the magnetization that can be explained by Curie’s law and by a slight oxidation of the NWs. Compared to previous findings for the oxidation of bare NWs, the result shows that the PDMS considerably slows down the oxidation of NWs but does not completely avoid it. This issue could be addressed by using single crystalline iron NWs, in which only the shell oxidizes and the iron core is retained up to 150 °C [24]. The spin-valves provide a stable MR performance up to around 200 °C, at which point the magnetic order of pinned and reference layer are lost.

Overall, the performance of the tactile sensor remains viable at high temperatures and operation to at least 140 °C is possible with the potential to go up to 200 °C. The sensor operation at elevated temperatures comes with a trade off in the sensitivity and signal amplitude. At 140 °C the sensitivity is reduced to 54% of the one at room temperature. This is caused by the reduction in magnetization of NWs and the stiffening of the polymer.

## Figures and Tables

**Table 1:** Tactile sensor results at different temperatures. Pressures are calculated by considering the forces acting on the sensor’s active area.

Temperature (°C)	Operating Range (mN) or (kPa)	Sensitivity (Ω/mN) or (Ω/kPa)	Maximum ΔR @ 15 mN (Ω)	Resolution (mN) or (kPa)	Noise Level ± (Ω)
20	0–15.0	46	700	1.31	30
50	0–16.2	39	581	1.69	33
80	0–16.5	35	531	2.17	38
110	0–17.1	29.4	462	2.85	42
140	0–17.4	25	385	3.91	49

## Abbreviations

The following abbreviations are used in this manuscript:

NWs: Nanowires
GMI: Giant magnetoimpedance
GMR: Giant magnetoresistive
PDMS: Polydimethylsiloxane
SDS: Sodium dodecyl sulfate
PMMA: Poly (methylmethcrylate)
